# Global Human Appropriation of Net Primary Production for Biomass Consumption in the European Union, 1986–2007

**DOI:** 10.1111/jiec.12238

**Published:** 2015-02-17

**Authors:** Thomas Kastner, Karl‐Heinz Erb, Helmut Haberl

**Keywords:** biomass, consumption‐based accounts, displacement of land use, human appropriation of net primary production (HANPP), industrial ecology, telecoupling

## Abstract

The ongoing globalization process strengthens the connections between different geographic regions through trade. Biomass products, such as food, fiber, or bioenergy, are increasingly traded globally, thereby leading to telecouplings between distant, seemingly unrelated regions. For example, restrictions for agricultural production or changes in bioenergy demand in Europe or the United States might contribute to deforestation in Latin America or Sub‐Saharan Africa. One approach to analyze trade‐related land‐use effects of the global socioeconomic biomass metabolism is the “embodied human appropriation of net primary production” or eHANPP. eHANPP accounts allocate to any product the entire amount of the human appropriation of net primary production (HANPP) that emerges throughout its supply chain. This allows consumption‐based accounts to move beyond simple area‐demand approaches by taking differences in natural productivity as well as in land‐use intensity into account, both across land‐use types as well as across world regions. In this article, we discuss the eHANPP related to the European Union's (EU) consumption of biomass products in the period 1986–2007, based on a consistent global trade data set derived from bilateral data. We find a considerable dependency of the EU on the appropriation of biological productivity outside its own boundaries, with increasing reliance on Latin America as a main supplier. By using the EU as an illustrative example, we demonstrate the usefulness of eHANPP for assessing land‐use impacts caused by nations’ socioeconomic activities and conclude that the eHANPP approach can provide useful information to better manage ecosystems globally in the face of an increasingly interconnected world.

## Introduction

The volume of traded agricultural products is rising faster than the global production of biomass in agriculture and forestry (FAO 2014), thereby increasingly affecting global patterns of land use and land‐use intensity (Meyfroidt et al. 2010; Kastner et al. 2014). The increasing number of trade links between countries and rising trade volumes, in particular, those of biomass‐related products, result in a growing spatial disconnect between the places of production and the places where the products are being consumed (Erb et al. 2009). Consequently, the importance of trade‐related telecouplings—that is, interdependencies between distant, seemingly unrelated regions—is growing (Güneralp et al. 2013; Seto et al. 2012; Liu et al. 2013). In particular, there are concerns that measures or policies aiming at the conservation of forests or other environmental assets in one region may result in displacement of agricultural production or other activities to other regions (Meyfroidt et al. 2010). Discussions on how to account for such leakage effects in the context of REDD (reduced emissions from deforestation and forest degradation) policies provide an example (e.g., Henders and Ostwald 2014). Comprehensive assessments of land‐use–related environmental impacts from a consumer perspective is one of the frontiers of socioeconomic metabolism research.

Different approaches have been proposed to account for the land demand of production and consumption activities. Most are based either on unweighted accounts of the area of cropland, pasture, and forests (Van Vuuren and Smeets 2000; Gerbens‐Leenes et al. 2002; Erb 2004; Yu et al. 2013; Kastner et al. 2014) or on areas weighted by some measure of productivity, for example, the “global hectares” approach as used in the ecological footprint approach (e.g., Steen‐Olsen et al. 2012; Weinzettel et al. 2013 based on Wackernagel and Rees 1996). Though there are advantages of using area units, among others, their good communicability, the problem remains of how to take differences in natural productivity, as well as in land‐use intensity, into account. Such differences exist both across land‐use types and across different world regions or nations. An approach that can tackle such differences is built on a system‐level indicator of land‐use intensity (Erb et al. 2013; Kuemmerle et al. 2013), the “human appropriation of net primary production” or HANPP (Vitousek et al. 1986; Imhoff et al. 2004; Haberl et al. 2007). HANPP indicates how much of the potential productivity of ecosystems is appropriated by humans, either because replacement of pristine ecosystems with human‐modified landscapes results in changes in net primary production (NPP; this change in annual NPP resulting from land use and land conversion is abbreviated HANPP_luc_) or because NPP is removed from ecosystems to serve human purposes, such as food, feed, fiber, or bioenergy production (biomass harvest denoted as HANPP_harv_; for detailed definitions and concepts, see the *Methods and Data* section below and refer to Haberl et al. [2014]).

The embodied HANPP approach provides a link between approaches that account for the biophysical basis of socioeconomic activities and accounts of ecological consequences of land use. Embodied human appropriation of net primary production (eHANPP) accounts provide information on how much HANPP occurs along supply chains of biomass products and can thus give a comprehensive picture of HANPP impacts of biomass production, trade, and consumption, covering both the HANPP_harv_ and the HANPP_luc_ components. Whereas earlier eHANPP assessments were based on a net trade approach covering relatively few products (e.g., Erb et al. 2009), a number of issues call for the use of bilateral trade data sets covering traded products comprehensively, when aiming for consumption‐based accounts of land‐use impacts (Kastner et al. 2011b; Haberl et al. 2012a). Most important, owing to the heterogeneity of the resource land and its use, information on the location of land‐use activities, such as crop cultivation or wood extraction, is crucial. Comprehensive accounts therefore have to tackle, among others, the following issues: (1) the re‐export problem, that is, the need to trace back products to the place where the extraction of a primary product (e.g., soybeans, timber, or grass) is harvested, which is challenging for products with complex global supply chains (Kastner et al. 2011b); (2) the conversion problem, that is, the fact that products may undergo far‐reaching transformations during production chains, for example, animal products such as meat, milk, or eggs derived from grazing, haymaking, and fodder crops, but also food crops; and (3) the necessity to quantify the land‐use–related changes in annual NPP associated with biomass production.

We here present results of an empirical analysis of the eHANPP related to the European Union's (EU; see the *Methods and Data* section below for details on region definitions) biomass metabolism—that is, the consumption of biomass products (plant‐ and animal‐derived food as well as fiber crops and wood) consumed within the EU. Based on physical bilateral trade data and global data sets on HANPP, biomass metabolism, and land use, we establish a time series for the period from 1986 to 2007. We use the results to analyze and locate displacement effects related to the EU's consumption and trade patterns, including their changes in the last two decades. To highlight its usefulness for providing an integrated measure of land‐use impacts, we compare the eHANPP approach to values of purely area‐based approaches. We also explore the value of the concept for assessing resource‐use intensities by relating eHANPP to data on monetary trade flows.

## Methods and Data

### Consumption‐based Accounts of HANPP

HANPP represents a comprehensive measure of land‐use intensity (Erb et al. 2013), accounting for human alteration in biomass availability in ecosystems through land‐use practices. To do so, prevailing NPP levels after harvest activities in managed or used ecosystems are compared to hypothetical NPP values of a reference system without human land‐use activities, that is, the NPP of the potential vegetation (NPP_pot_; in our data derived from the global digital vegetation model LPJmL [Bondeau et al. 2007]; for details, see Haberl et al. [2007]; Krausmann et al. [2013]). The approach integrates two effects land use has on annual NPP flows in terrestrial ecosystems: (1) Harvested biomass (HANPP_harv_) is defined comprehensively in our data set, that is, considering biomass flows that are used as socioeconomic resources (domestic extraction) as well as all biomass killed in the harvest process, but not further used (e.g., roots killed through harvest). This definition provides a comprehensive measure of how harvest activities alter NPP flows in ecosystems; depending on the research question, this definition can be adapted (e.g., when looking at changes in ecosystem carbon stocks, only the biomass leaving the ecosystem might be relevant; for discussions on the implications of differences in the inclusiveness of the definition, refer to Haberl et al. [2014]). (2) Human‐induced changes in ecological productivity of managed land: for instance, if highly productive forest ecosystems are replaced by less‐productive agro‐ecosystems, the difference in annual NPP flows of the two is included in the calculated HANPP values and referred to as HANPP_luc_ (Haberl et al. 2014; note that, in intensively managed land systems, HANPP_luc_ can become negative, if actual NPP levels rise above potential ones). Thus, three different flows of NPP in ecosystems are differentiated in a HANPP approach: (1) potential NPP; (2) actual NPP; and (3) NPP remaining after harvest, with HANPP being defined as the difference between (1) and (3) or the sum of HANPP_harv_ and HANPP_luc_.

Whereas traditional HANPP accounts are production based, that is, assess HANPP for the location (or territory) where biomass is produced and land use takes place, we here establish *consumption‐based accounts* (CBA). Such accounts calculate the HANPP “embodied” in final biomass products (Erb et al. 2009; Haberl et al. 2009, 2012a). In our CBA, we assess the amount of HANPP embodied in the consumption of products from agriculture and forestry (i.e., biomass products) within individual countries.^1^ We are able to differentiate these eHANPP flows into a component originating from direct domestic production, a component of imports from EU countries, and a component of imports from outside the EU. Further, we contrast CBA with *production‐based accounts* (PBA) broken down into eHANPP related to domestic consumption and eHANPP for biomass exports. Owing to our focus on the EU, we further differentiate the trade component into exports to other EU countries and into exports to non‐EU countries.

For our eHANPP data set, we rely on data sets for biophysical trade flows of forestry (Kastner et al. 2011a; refer to Table 1 in that publication for the definition of wood products included) and agricultural products (Kastner et al. 2014; refer to Table S3 in that publication for primary products included), which, in turn, are largely based on FAOSTAT trade and production data (FAO 2014). These data sets establish—following the above‐described accounting logic—production‐ and consumption‐based accounts as well as global international trade relations in terms of primary wood and crop equivalents. For instance, trade in paper was converted into trade in primary wood equivalents and trade in soybean oil was converted into primary soybean equivalents (for details, refer to Kastner et al. [2011a, 2014]). The data also include comprehensive estimates of feed crops embodied in the traded animal products (Kastner et al. 2014) and address the problem of re‐exports to be able to establish clear links between country of production and country of consumption, omitting countries where only processing steps took place (for details and formulae on the algorithm used, see Kastner et al. [2011b]).

**Table 1:** Biomass flows^a^ and eHANPP of the EU (EU‐27) in 2007 compared to other world regions for both consumption‐based accounts (CBA) and production‐based accounts (PBA) accounts

	*Biomass flows* a		*eHANPP flows*		*eHANPP intensity*	
	*(t dm/cap/yr)*		*(t dm/cap/yr)*		*(t HANPP/t biomass)*	
	*CBA*	*PBA*	*CBA*	*PBA*	*CBA*	*PBA*
North America	1.9	2.4	7.7	9.4	4.1	3.9
***EU‐27***	***1.2***	***1.0***	***5.3***	***4.5***	***4.5***	***4.3***
Oceania	1.1	1.9	11.3	22.3	9.9	12.0
South America	1.0	1.4	9.3	11.1	9.4	8.0
FSU and other Europe	0.9	1.0	6.6	6.7	7.5	6.7
Central America and Caribbean	0.7	0.5	5.9	4.5	8.4	9.9
Southeast Asia	0.7	0.8	4.7	4.5	7.0	5.9
Northern Africa and Western Asia	0.6	0.3	2.7	2.1	4.9	6.7
Eastern Asia	0.5	0.4	2.1	1.7	3.9	3.9
Sub‐Saharan Africa	0.4	0.3	6.0	6.0	15.9	17.4
Southern Asia	0.4	0.4	1.8	1.9	4.9	5.3
World	0.7	0.7	4.0	4.0	6.1	6.1

Note

The last two columns show the HANPP intensity expressed as tonnes of eHANPP per tonne of primary equivalent biomass flow. Region definitions other than the EU‐27 follow Kastner and colleagues (2014)

a biomass expressed in primary crop and wood equivalents, details see text.

eHANPP = embodied human appropriation of net primary production; HANPP = human appropriation of net primary production; EU = European Union; FSU = Former Soviet Union; t = metric tonnes; dm = dry matter; cap = capita; yr = year.

### eHANPP Factors

For our global eHANPP data set, we combine data from the extensive databases on global patterns and trends in HANPP from a production perspective (one for the year 2000 [Haberl et al. 2007]) and one spanning from 1910 to 2005 (Krausmann et al. 2013) with data sets on international trade and consumption of biomass products (Kastner et al. 2011a, 2014). For details on these data sets and their establishment, the interested reader is referred to the original publications. Here, we describe how we combined these data sets to address the third and final challenge outlined in the introduction: What amount of HANPP is embodied in one unit of primary crop or wood equivalent, including both the HANPP_harv_ and HANPP_luc_ components? In our analysis, we use metric tonnes of dry matter biomass per year (t dm/yr) as the unit of the annual flows of NPP, HANPP, and eHANPP (all of which could also be measured as carbon or energy flows). For instance, eHANPP related to 1 tonne (t) of traded soybean oil would include the sum of the (1) production of soybean equivalents needed to produce the oil, (2) amount of plant biomass other than the primary crop product (soybeans) killed in the harvest process, and (3) amount of HANPP_luc_ induced by conversion of the potentially prevailing vegetation at the respective location of crop production to cropland growing soybean with the prevailing productivity, all expressed as annual flows.

The method to establish eHANPP factors differs for the main land‐use types: For grassland and HANPP related to the cultivation of fodder crops, we assign the entire national‐level HANPP flows of the respective land‐use types (from Krausmann et al. 2013) to the ruminant products (and traded derived products) covered in Kastner and colleagues (2014). This allows deriving factors for, for instance, the amount of grassland HANPP embodied in 1 t of beef produced in a certain country. eHANPP related to industrial roundwood products is only included from 1997 onward (in both CBA and PBA), owing to unavailability of bilateral trade‐flow data before that year. Here, we use data directly from Kastner and colleagues (2011a) because these include the wood products themselves, plus estimates on bark and on logging damage; this corresponds to the definition of HANPP on forestlands. This way, we arrive at national‐level factors of eHANPP linked to the production of one unit of primary equivalent wood product.

For croplands, we chose a more complex approach, owing to their relevance in both overall HANPP levels (e.g., Haberl et al. 2007) and overall agricultural trade. In a first step, we establish national‐level values for the potential NPP (the reference value in the HANPP approach) of areas sown to different crops; this accounts for the possibility that the average land quality may differ between individual crops. We establish these factors by overlaying data on the spatial distribution of crop cultivation (from Monfreda et al. 2008) with a global map of potential NPP (from Haberl et al. 2007). To arrive at crop‐specific eHANPP factors, we combine these potential NPP values with national‐level values of HANPP as the percentage of potential NPP for cropland (from Krausmann et al. 2013). Thereby, we establish crop‐specific factors of HANPP embodied per unit crop primary equivalent produced for over 140 crops and over 200 nations (e.g., the amount of HANPP linked to one unit soybean equivalent).

### Putting the Pieces Together

With the eHANPP factors for grassland, forests, and cropland and the data sets on primary crop and wood equivalents, we establish consumption‐based accounts of eHANPP at the global level, covering over 200 nations. In this analysis, we focus on the EU, where we look at regional totals as well as values of the individual member states (in this article, we include 27 states in the EU region throughout time; regions other than the EU‐27 follow Table S2 in Kastner et al. [2014]). The consumption‐based account of global eHANPP flows covers approximately 90% of the global total HANPP in the year 2000 (Haberl et al. 2007). We do not include human‐induced vegetation fires and HANPP resulting from the establishment of infrastructure and settlement area, mainly because it is conceptually not clear how to allocate these HANPP components to biomass trade flows.

Where available, we utilize annual data to establish a time series from 1986 to 2007. HANPP values are available for time cuts only (1980, 1990, 2000, and 2005). We therefore interpolate linearly values between the available years where necessary and extrapolate linearly for the years 2006 and 2007.

We employ the amount of eHANPP per unit of primary wood or crop equivalent, or HANPP intensity, as the measure of land‐use efficiency (in the example on soybean oil above, it would be [a + b + c] divided by [a]). A high HANPP intensity value implies large impacts on ecosystems per unit of socioeconomically valuable output (e.g., cropping with low output per unit area); a low value indicates lower impacts per unit output. To augment our biophysical analysis, with socioeconomic data we use bilateral monetary trade data for agricultural products (from FAO 2014), which we aggregate the same way as the physical data sets (including the same set of products and countries).

## Results and Discussion

### Overview: eHANPP Trends in the European Union from Production and Consumption Perspective

**Figure 1:**
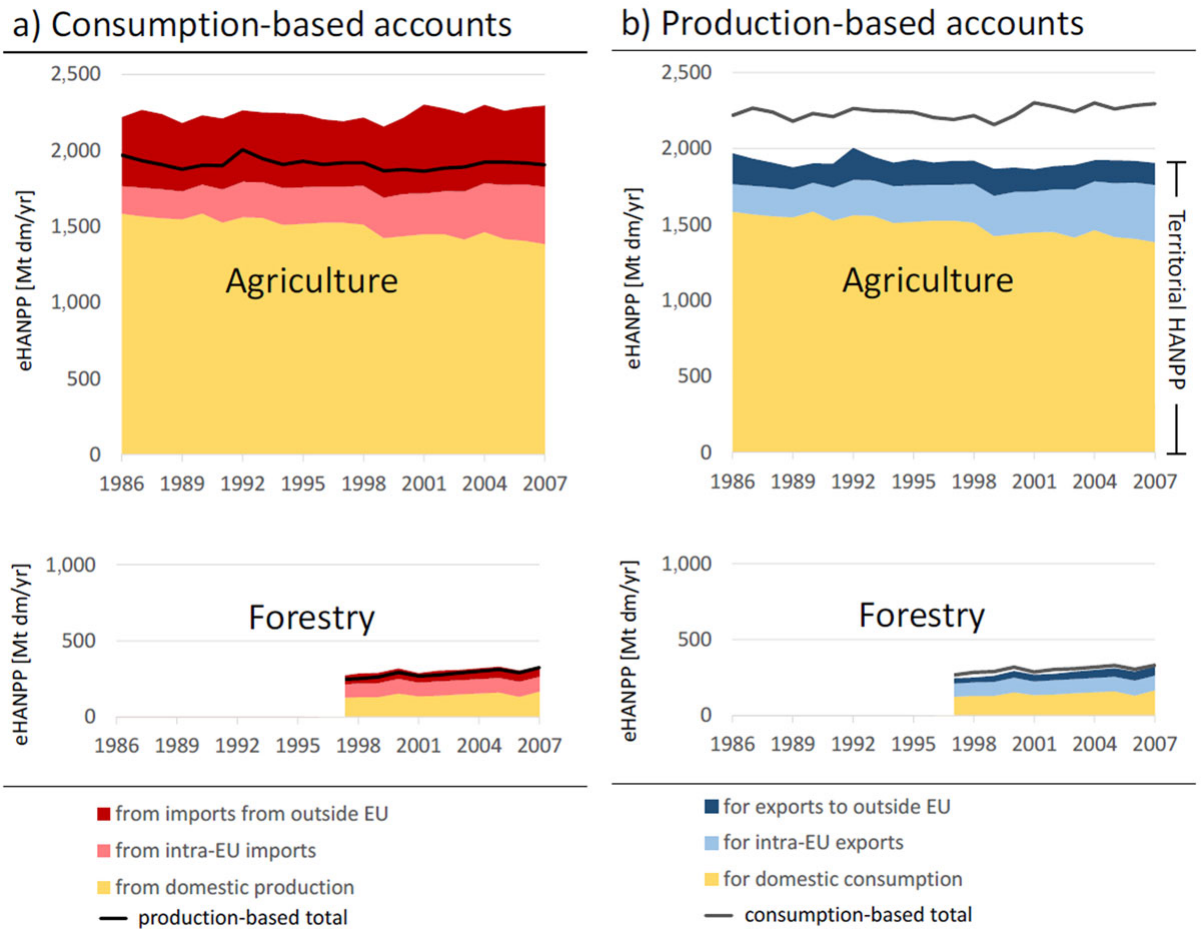
eHANPP in the European Union, 1986–2007. (a) Consumption‐based accounts and (b) production‐based accounts. Results are displayed separately for eHANPP related to agriculture (cropland and grazing lands) and forestry (forestlands), because data for the latter were available only from 1997 onward. eHANPP related to trade is differentiated between trade among EU countries and trade of the EU with the rest of the world; the exact numbers are provided in tables S1 to S4 in the supporting information available on the Journal's website. eHANPP = embodied human appropriation of net primary production; HANPP = human appropriation of net primary production; Mt dm/yr = million tonnes dry matter per year; EU = European Union.

The embodied HANPP related to biomass products consumed within the EU increased slightly throughout the study period (figure 1a; for exact numbers, refer to the Supporting Information on the Web). Figure 1 displays the results separately for agriculture and forestry resulting from the different temporal coverage of the two. Comparing the two components reveals the dominance of the former, amounting to approximately 9 times of the eHANPP of forestry in the CBA. In the agricultural part of the CBA, the contribution from production on own territory within the EU countries declined from approximately 1.6 gigatons of dry matter biomass per year (Gt dm/yr) in 1986 to approximately 1.4 Gt dm/yr by 2007. eHANPP related to products consumed within the EU countries from imports increased throughout the study period, with eHANPP related to imports countries sourced from outside the EU larger than that sourced from inside the EU, albeit the latter exhibiting larger growth rates.

Contrasting CBA and PBA (figure 1) reveals that the EU was a net importer in terms of eHANPP throughout the study period. eHANPP related to exported agricultural products (figure 1b) grew as well, but, in this case, exports to other EU countries play a much larger role than exports to the rest of the world, which declined slightly over time (figure 1b).

Our results underline the growing role of international trade for national and regional supply with biomass products. In 1986, approximately 29% of the eHANPP related to the domestic consumption of biomass products in the EU's member states stemmed from imports; this number grew to 41% in 2007. Net imports (eHANPP related to import minus eHANPP related to export) amount to approximately 10% of the CBA eHANPP. For agricultural products, measured in units of eHANPP, the volume of imports exceed exports by a factor of just above 2 in 1986; this factor increased to almost 4 by 2007. This indicates that the EU depends, to a considerable and growing extent, on productive capacities of ecosystems outside its boundaries, despite the high productivity achieved on its own territory (Haberl et al. 2012b). However, this increase in eHANPP of net imports linked to agricultural trade was not linear: In the first half, a slight decline occurred, whereas in the second decade a considerable increase in net imports was observed. Even with the strong economic integration and free trade within the EU, in 2007 eHANPP flows for imports into the EU for domestic consumption were approximately 40% larger than the sum of all eHANPP flows related to intra‐EU biomass trade.

### Changes in Regional Patterns of eHANPP Related to Trade

**Figure 2:**
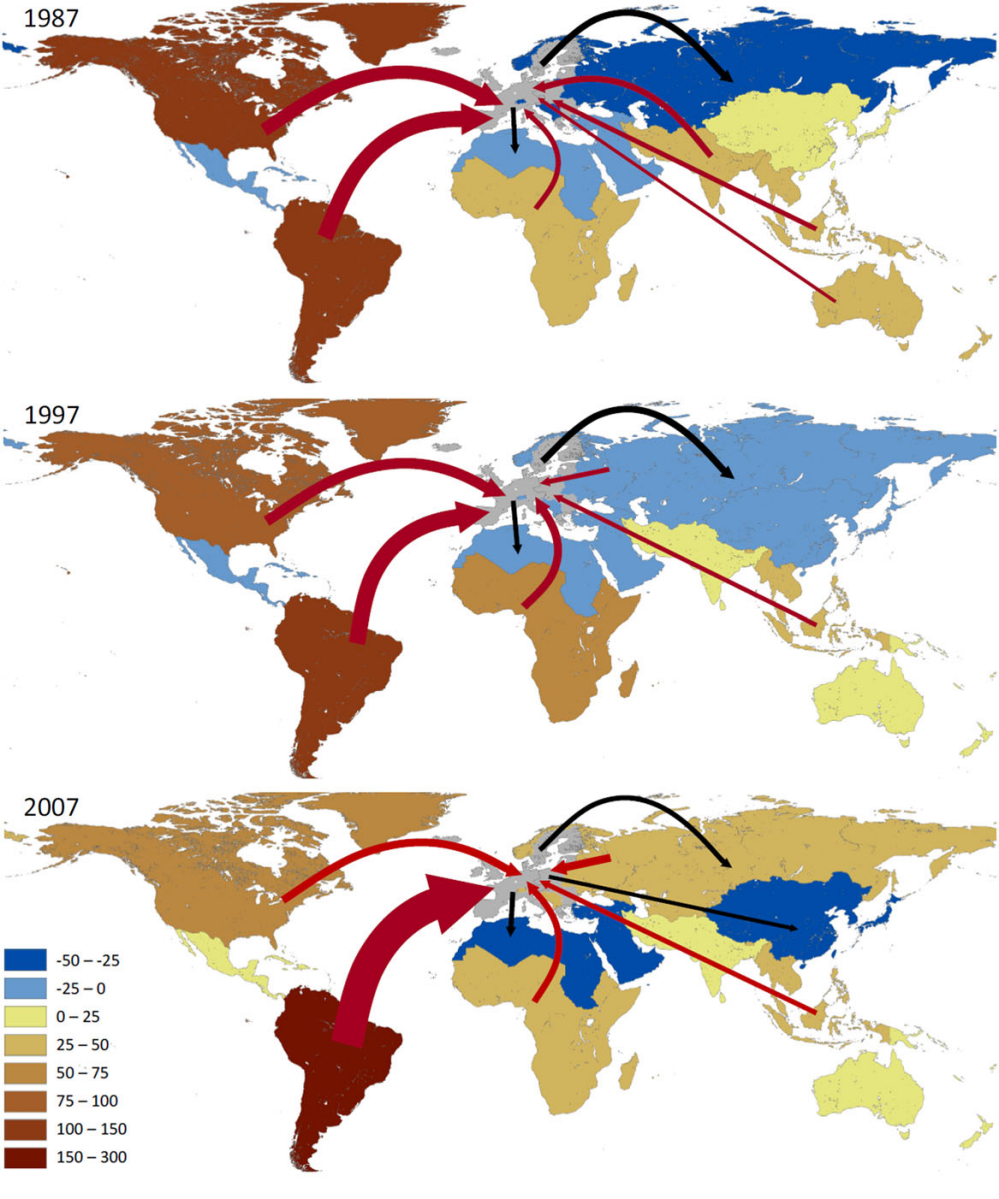
Maps of the net trade balance of HANPP embodied in the trade of biomass products between the EU and ten other world regions, measured in tonnes dry matter of eHANPP per year. Regions in reddish colors are net exporters of eHANPP to the EU; regions in bluish colors are net importers of eHANPP. Arrows indicate the largest gross flows, eHANPP flows exceeding 30 Mt dm/yr; red arrows refer to HANPP embodied in the EU imports, and black arrows to HANPP embodied in EU exports; the size of the arrows are according to scale, with the largest flow being the one from South America in 2007 (286 Mt dm/yr); the exact numbers for all years are provided in tables S5 to S6 in the supporting information on the Web. eHANPP = embodied human appropriation of net primary production; HANPP = human appropriation of net primary production; Mt dm/yr = million tonnes dry matter per year; EU = European Union.

Throughout the study period, the largest flows of eHANPP related to trade stem from North and South America, as shown in maps of the eHANPP related to the EU foreign trade in 1987, 1997, and 2007 (figure 2). The maps show the net trade balance of the EU with a number of other regions in 1987, 1997, and 2007. Throughout the period, South America strongly gains and North America is gradually losing relevance in providing ecosystem services linked to the EU's consumption. EU biomass imports were also related to considerable eHANPP flows in Sub‐Saharan Africa and South‐East Asia. Throughout the study period, the EU was a net exporter in terms of eHANPP to Central America, North Africa, and West Asia. Though the EU was also an eHANPP net exporter to the region of the Former Soviet Union and other European regions in 1987, this trend reversed in recent years and the EU's imports from this region now outweigh its exports (see, e.g., Schaffartzik et al. [2014] on the EU's increasing biofuel imports from the Ukraine).

The results clearly demonstrate that the supply of the EU with biomass products has a global footprint that is increasingly focused on one of the two regions of the world with substantial potentials for cropland expansion: Latin America (FAO and IIASA 2000; Ramankutty et al. 2002). The other region with abundant cropland potentials, Sub‐Saharan Africa, is also found to be a net exporter of eHANPP to the EU, but at a much lower level. In general terms, our findings corroborate earlier results showing that imports are sourced predominantly from regions with a low population density (Erb et al. 2009; Kastner et al. 2011a; Haberl et al. 2012b).

### Regional Differences in Land Use and Biomass Consumption

One particularly interesting question is how biomass flows and eHANPP are related, given that eHANPP accounts are, to a large degree, influenced by the efficiency of land use in a region. The comparison of flows of commercially valuable biomass and eHANPP allows analyzing and comparing land‐use efficiencies of biomass production/consumption systems in different world regions (table 1). Biomass flows in table 1 are expressed as primary biomass equivalents (i.e., commercially valuable biomass harvest used for further processing) and thus required as input for considered derived products. The ratio of eHANPP to primary biomass flows depends on two factors: (1) HANPP_luc_ per unit of biomass produced. A large HANPP_luc_ value will result in a high eHANPP/biomass‐flow ratio, indicating that managed ecosystems are less productive than potential vegetation. (2) The ratio of total biomass harvest as accounted for in the HANPP method (denoted as HANPP_harv_) to the amount of commercially valuable primary biomass products. This ratio provides information on the fraction of biomass affected during land use that is actually put to use. A considerable fraction of biomass is destroyed during harvest and not recovered and used for production (e.g., residues, roots of plants killed during harvest, biomass consumed in human‐induced vegetation fires, and so on).

The efficiency with which primary biomass is converted to final products is not visible in these numbers because the final fate of primary biomass flows depends on the structure of the prevailing production/consumption systems in the respective regions. A high per capita biomass flow in primary crop equivalents is indicative of consumption patterns in which products with large primary biomass flows per unit of final product, above all, animal food products, play a prominent role.

Table 1 relates per capita biomass flows to eHANPP in both consumption‐ and production‐based accounts for 11 world regions. The EU ranks second (after North America) among 11 world regions in per capita biomass flows according to CBA, but only fourth (equal with Oceania) according to PBA. In contrast, when ranked according to per capita eHANPP values, the EU ranks only eighth in the PBA (seventh in CBA). This difference in ranking indicates that—from a HANPP perspective—the EU's biomass metabolism is very efficient. The fact that the EU's HANPP intensity (HANPP, respectively, eHANPP per unit of biomass flow) is slightly higher in the consumption than in the production perspective results from the fact that yields are higher and losses lower in the EU than in the countries from which it imports. Given that efficiencies in terms of HANPP are often the result of high external inputs into agriculture (e.g., inorganic fertilizers), complementary consumption‐based indicators are required to draw up a comprehensive picture of land‐use impacts (e.g., Leach et al. 2012; MacDonald et al. 2012; Leip et al. 2013).

Per capita levels of biomass consumption are considerably higher in North America than in the EU, whereas it is slightly lower in Oceania and South America, both of which surpass the EU in the production perspective (table 1). This is related to the strongly export‐oriented structure of these regions, which are both relatively sparsely populated and have relatively inefficient biomass production systems in terms of HANPP (Krausmann et al. 2013).

### Differences in the eHANPP Balances of Individual European Union Countries

**Figure 3:**
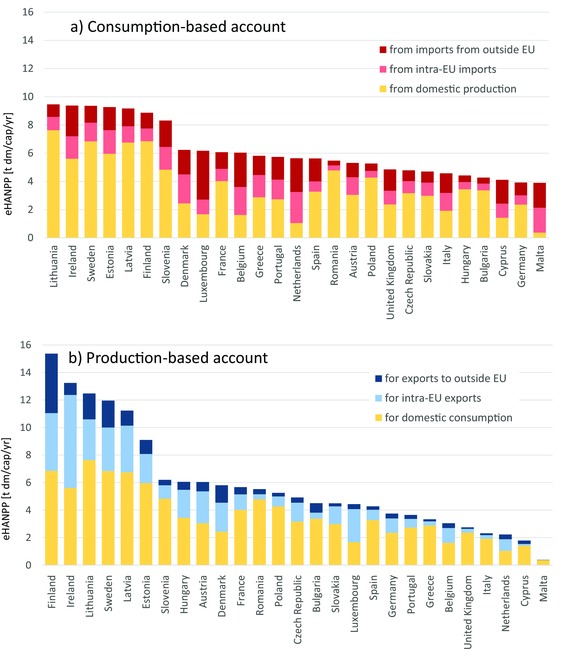
Per capita values of eHANPP in the EU's member states in 2007. (a) Consumption‐based account and (b) production‐based account. Countries are ranked from highest to lowest per capita value in the respective accounts; the exact numbers are provided in tables S7 to S8 in the supporting information on the Web. eHANPP = embodied human appropriation of net primary production; t dm/cap/yr = tonnes dry matter per capita per year; EU = European Union.

Figure 3 presents per capita results for individual EU countries for the year 2007. Countries are ranked according to the CBA (figure 3a) and the PBA (figure 3b), with the same subcomponents distinguished as in figure 1. In both accounts, values are highest in sparsely populated regions, such as the Scandinavian and Baltic countries as well as Ireland. They are lower in the densely populated Central and Southern European countries. Countries with low population density are also the largest exporters in terms of eHANPP, with Finland, Ireland, Sweden, Lithuania, and Latvia showing especially high values, with up to half of their territorial HANPP associated with export of biomass products. On the other hand, the CBA of countries such as Malta, the Netherlands, Denmark, Luxembourg, or Belgium is dominated by eHANPP imports to a large degree. For countries such as Austria, and to a certain degree also France, the domestic, exports, and imported components are of a similar magnitude.

Comparing the rankings in figure 3a and 3b (CBA vs. PBA) reveals large differences and thus further highlights the relevance of trade in the EU: Whereas strongly net importing countries, such as the Netherlands and Belgium, move up more than ten places in the CBA ranking, net exporting countries, such as Hungary, Bulgaria, and Austria, are found further down in the CBA compared to the PBA ranking. When looking at per capita (cap) values, the range is much larger in the PBA ranking (from more than 15 t dm/cap/yr in Finland to less than 1 t dm/cap/yr in Malta) compared to the CBA (here, the maximum and minimum differ by a factor just over 2).

### Trade Seen through Biophysical and Monetary Lenses

Table 2 displays the EU's trade in agricultural products in physical (eHANPP and biomass flows) and monetary terms, differentiating intra‐EU trade from trade between the EU and the rest of the world. Looking at the latter, the numbers reveal that the EU is a net importing (physical trade balance = imports minus exports) region, both in terms of primary biomass flows and eHANPP. Though the monetary trade balance (calculated as exports revenues minus the costs of import) for the considered agricultural products is slightly negative, the picture is much more balanced in terms of monetary, than in terms of biophysical, data. For instance, in 2007, imports in terms of eHANPP outweigh exports by a factor of 3.7 for eHANPP and 2.5 for biomass flows; in monetary terms, this ratio was only 1.2.

**Table 2:** Trade of the EU in terms of eHANPP, biomass flows (in primary equivalents), and monetary flows

	*Imports from outside EU*			*Exports to outside EU*			*Intra‐EU trade*		
	*1987*	*1997*	*2007*	*1987*	*1997*	*2007*	*1987*	*1997*	*2007*
eHANPP (Mt dm/yr)	511	428	535	177	157	144	187	236	376
Biomass flows^a^(Mt dm/yr)	71	62	96	38	39	38	38	54	76
Monetary flows^b^(BN $/yr)	42	55	95	27	49	81	69	117	252
eHANPP intensity (kg dm/$)	12.2	7.8	5.6	6.5	3.2	1.8	2.7	2.0	1.5
Biomass intensity (kg dm/$)	1.7	1.1	1.0	1.4	0.8	0.5	0.6	0.5	0.3

*Sources*: Own calculations and FAO (2014).

*Note*: Trade flows are split into the EU's imports from outside the EU, the EU's exports to outside the EU, and intra‐EU trade (where imports = exports per definition). The former two constitute the EU's trade balance. Monetary flows in current US$. Monetary resource intensities in terms of eHANPP and biomass flows per monetary unit are shown.

a Traded biomass flows expressed as primary crop and wood equivalents; for details, see text.

b One BN $ = 10^9^ $.

EU = European Union; eHANPP = embodied human appropriation of net primary production; Mt = million tonnes; dm = dry matter; yr = year; BN = billion; kg = kilograms.

The observed differences are linked to the higher intensities per monetary unit of imports: For instance, in 2007, one dollar of costs for imports was linked to 3 times the eHANPP than per dollar export revenue; over time, this disparity widened as the corresponding factor had been below 2 in 1987. A main reason for these differences could be the fact that the EU imports relatively unprocessed bulk products and exports higher processes products (FAO 2014). Comparing values for intra‐EU trade with the trade flows to and from the rest of the world, the former dominate from a monetary perspective, exceeding the absolute value of the trade of the EU with the rest of the world by a factor of 1.4 in 2007. From an eHANPP perspective, this factor was below 0.6 in 2007, indicating that intra‐EU trade here (despite its fast growth rates) still plays a relatively minor role.

Overall, comparing these aggregated numbers and intensities shows that the EU benefits disproportionally in monetary terms from agricultural trade whereas ecological impacts of trade, in terms of eHANPP, occur to a disproportionally large share outside the EU. More in‐depth research into relations between biophysical and monetary accounts of bilateral trade will be an interesting area for follow‐up studies.

### A Comparison of eHANPP with a Land Area Approach

**Figure 4:**
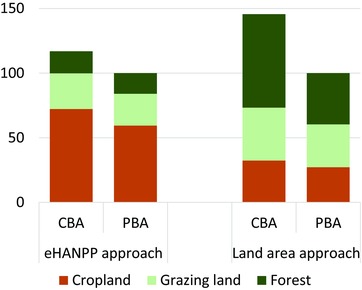
The European Union's biomass metabolism in both consumption‐ and production‐based accounts (CBA, PBA) using the eHANPP metric as well as an unweighted area approach. Values are indexed to 100 for the respective total according to the PBA. Areas were calculated using factors derived from Haberl and colleagues (2007). eHANPP = embodied human appropriation of net primary production.

One of the drawbacks of evaluating trade‐related effects based on area demand alone is that land can be used with very different intensities, both within one land‐use class (e.g., different stocking densities of livestock on intensively and extensively grazed land) and even more so between land‐use classes (e.g., between cropland, grazing land or forestry). Ecological impacts of land use, however, are closely linked to the intensity of use. This calls for alternatives to simple area‐demand metrics. With its focus on ecological energy flows, the basis of many ecosystem services (Millennium Ecosystem Assessment 2005), eHANPP accounts allow to integrate land‐use pressures consistently across different land‐use types. The differences between trade‐related accounts based on eHANPP and those based on area are quite interesting (figure 4).

In the eHANPP approach, cropland accounts for more than half of the total impact according to both CBA and PBA (figure 4). In the unweighted area‐demand approach, the relative importance of cropland is much lower. The reason is that grasslands and forests are, on average, used much less biomass intensively than croplands. Both the impact of land conversion on annual NPP flows (as measured as HANPP_luc_) and the amount of harvest (HANPP_harv_) per unit of area are typically highest on cropland. This is reflected in the dominance of cropland in the eHANPP results. In forests, output per unit area is bound to be low because trees allocate only a limited fraction of their total biomass production to long‐lived structures that are available for harvest after several decades of growth (Körner 2009; Schulze et al. 2012). The intensity with which grasslands are used varies immensely between different systems and regions (Herrero et al. 2013); however, in virtually all cases, the intensity with which grasslands are used is much lower than that of croplands.

It is also striking that the difference between CBA and PBA is much larger when evaluated with an unweighted area approach (figure 4). This results largely from the forestry sector: European forests are used more intensively than the forests in many of the countries from which the EU imports (especially Russia), that is, a relatively smaller area of forest is required to produce one unit of wood within the EU than it is on average in countries from which the EU sources it imports. The overall appropriation of forest NPP is, however, similar (thus little difference between CBA and PBA in the eHANPP account).

Finally, it has to be noted that, whereas the (e)HANPP approach is able to capture a number of the many aspects of land‐use intensity (Erb et al. 2013), additional information will be needed to build a comprehensive picture of environmental consequences of land use. In particular, HANPP accounts are not able to depict input‐related intensity effects, such as, for instance, ecological consequences of fertilization, irrigation, or mechanization (see above).

## Outlook and Conclusions

In order to satisfy its demand for biomass for food, feed, fiber, and bioenergy, the EU depends, to a considerable and growing extent, on productive lands outside its own boundaries, thereby potentially displacing some of the environmental impacts related to its supply of biomass products to other countries or regions. If measured in units of the eHANPP, the EU's biomass imports exceed its exports by a factor of 3. The two world regions with substantial remaining cropland potentials, South America and Sub‐Saharan Africa, play a central role for satisfying the EU's demand for land‐related resources. South America alone supplies over 10% of the EU's entire eHANPP when measured in CBA. This corroborates earlier findings emphasizing the important role of trade in biomass products in enabling the EU's forest transition (Meyfroidt et al. 2010). The results also suggest that strong increases in bioenergy use within the EU would likely result in increased environmental pressures outside the EU, either directly, through increased imports of bioenergy carriers or their related feedstocks, or indirectly through increased food‐related imports (Searchinger et al. 2008; Lamers et al. 2011).

The issue of how to deal with growing telecouplings between geographic regions and their economies induced by international trade clearly presents a key frontier of socioeconomic metabolism research. The development of methods that are able to show contrasting perspectives on production/consumption systems, including their geographic characteristics and temporal dynamics, and provide us with consistent and meaningful results is of paramount importance. Considerable progress has been made in the last years with assessments of upstream resource requirements, as reflected in material flows (e.g., Bruckner et al. 2012; Wiedmann et al. 2013), energy flows (e.g., Kara et al. 2010) or greenhouse gas GHG emissions (Hertwich and Peters 2009; Davis and Caldeira 2010; Peters et al. 2011), and even biodiversity (Lenzen et al. 2012) and nutrient inputs into agriculture (Galloway et al. 2007; MacDonald et al. 2012). However, the development of robust accounting of land‐related upstream impacts of resource use is not straightforward, given that land in itself is a multidimensional resource. Its physical extent—the global terrestrial surface of approximately 130 million square kilomters—represents a planetary boundary that is not to be overcome. However, this absolute limit is of limited significance, given that land differs strongly in quality and intensity of use, and only some uses are mutually exclusive (e.g., crop production vs. carbon sequestration), whereas others can, to some extent, be combined in multifunctional landscapes (e.g., agricultural production and maintenance of valuable cultural landscapes). This renders upstream resource demand metrics that simply account for land areas used difficult to interpret.

The research presented in this article attempts to advance this research frontier by providing an illustrative case for the usefulness of the indicator, eHANPP. eHANPP focuses on a key element of ecological as well as socioeconomic dynamics: the net primary production, basis of all heterotrophic life, and a key resource for all societies. eHANPP concepts integrate two central aspects of land‐related impacts of resource use, that is, land quality (by its reference to the potential NPP) and land‐use intensity (by its focus on human‐induced alterations of NPP). In this manner, it moves beyond simple area‐based accounts that neglect these quality aspects of land (use). Because NPP—and, consequently, HANPP—is pivotal for biodiversity (e.g., Haberl et al. 2005) and for stocks/flows relations of ecosystem carbon, the eHANPP approach introduces a promising option to provide robust and comprehensive consumption‐based information on how socioeconomic activities affect—through their impact on biomass availability—ecosystems services. Such information is the prerequisite to tackle trade‐related land‐use impacts in an increasingly telecoupled world. By relating data on eHANPP to information on the economic value of biomass flows, we were able to point to the concept's usefulness when discussing resource intensities. Further investigation along these lines will produce valuable insights when aiming at limiting negative impacts per (monetary or physical) unit of useful output.

## Supporting information


**Supporting Information S1**: This supporting information contains eight detailed data tables underlying the three figures in the article.

## Footnotes

1 Note that, in contrast to other approaches (e.g., Bruckner et al. 2012; Moran et al. 2013; Wiedmann et al. 2013), the eHANPP approach does not cover indirect flows (e.g., flows related to the biomass contained in the machinery to produce bread). It does, however, include comprehensive estimates of the HANPP directly required for traded biomass products (e.g., HANPP associated with the cultivation of the wheat to produce 1 t of imported bread). See Haberl and colleagues (2009) for conceptual details.
